# Vibration Sensor Data Denoising Using a Time-Frequency Manifold for Machinery Fault Diagnosis

**DOI:** 10.3390/s140100382

**Published:** 2013-12-27

**Authors:** Qingbo He, Xiangxiang Wang, Qiang Zhou

**Affiliations:** 1 Department of Precision Machinery and Precision Instrumentation, University of Science and Technology of China, Hefei 230026, China; E-Mail: xifan@mail.ustc.edu.cn; 2 Department of Systems Engineering and Engineering Management, City University of Hong Kong, Tat Chee Avenue, Hong Kong, China; E-Mail: q.zhou@cityu.edu.hk

**Keywords:** vibration sensor, data denoising, time-frequency manifold, machinery fault diagnosis, bearing

## Abstract

Vibration sensor data from a mechanical system are often associated with important measurement information useful for machinery fault diagnosis. However, in practice the existence of background noise makes it difficult to identify the fault signature from the sensing data. This paper introduces the time-frequency manifold (TFM) concept into sensor data denoising and proposes a novel denoising method for reliable machinery fault diagnosis. The TFM signature reflects the intrinsic time-frequency structure of a non-stationary signal. The proposed method intends to realize data denoising by synthesizing the TFM using time-frequency synthesis and phase space reconstruction (PSR) synthesis. Due to the merits of the TFM in noise suppression and resolution enhancement, the denoised signal would have satisfactory denoising effects, as well as inherent time-frequency structure keeping. Moreover, this paper presents a clustering-based statistical parameter to evaluate the proposed method, and also presents a new diagnostic approach, called frequency probability time series (FPTS) spectral analysis, to show its effectiveness in fault diagnosis. The proposed TFM-based data denoising method has been employed to deal with a set of vibration sensor data from defective bearings, and the results verify that for machinery fault diagnosis the method is superior to two traditional denoising methods.

## 1. Introduction

Denoising has always been an important task in sensor data processing, and it has also become increasingly significant in the field of electronic measurement and instruments. Vibration sensor data from a mechanical system are often associated with important measurement information for machinery condition monitoring and fault diagnosis [1]. For example, vibration signals from defective rolling element bearings are generally observed as periodic transient impulses due to the rotating nature. Research has shown that these periodic transient impulses often reflect important physical information related to the machine dynamics. Effective analysis of the vibration signals is the basis of machinery fault diagnosis. However, in practice there always exists lots of background noise in collected vibration data, which will corrupt the fault-induced transient impulses. Hence, it is always an important aim to denoise the measured vibration signal and extract the intrinsic fault signatures for a reliable fault diagnosis.

Generally, data denoising can be conducted in either the time domain, or the frequency domain, or the time-frequency domain. In the time domain, a typical method is the time-domain averaging method which is most suitable for analyzing a strictly periodic signal [2]. In the frequency domain, a typical method is band-pass filtering, which only considers narrow band information [3]. Due to their transient properties, defect-induced vibration signals generally have a wide frequency band. Because the above two approaches can't take time and frequency information into account simultaneously, the information of transient impulses will be always lost or the noise will not be removed completely. On the contrary, the time-frequency representation can combine time and frequency information together, which can benefit data denoising with a synthetic consideration of both kinds of information [4]. By this approach, the noise in the entire time-frequency plane can be expected to be removed. Due to this advantage, time-frequency domain denoising approaches have been widely developed. Typical approaches are mainly based on the wavelet transform (WT) and the time-frequency analysis (TFA).

The WT has the merit of multi-resolution analysis, which is very suitable for detecting a transient state anomaly that is embedded in a normal signal. Hence, data denoising based on the WT is a research hotspot. Wavelet-based denoising methods are very popular at present [5–13]. However, some problems still remain. For instance, it is hard to select the optimal wavelet basis for signal denoising to avoid the loss of useful components in the signal, and there is no unique and effective method to choose the threshold value in discriminating the noise. The TFA has the merit that it can intuitively represent the time-frequency information in a two-dimensional domain, so it can be used to maintain good time-frequency properties in denoising. One of typical methods is the short-time Fourier transform (STFT) threshold denoising (also called spectrum subtraction), which has been popularly used for speech signal denoising [14]. There are still some remaining issues to be studied for this method, which makes the denoising effect unsatisfactory in complex noise background situations. A time-frequency domain averaging method to clean up the noise by calculating the geometric average in the time-frequency domain for a strictly periodic vibration signal was reported in [15]. There is also a study addressing threshold denoising in the reconstruction of a composite dictionary (combining impulse time-frequency dictionary and Fourier dictionary) multi-atom matching decomposition [16]. In the TFA-based denoising approach, one of the most important issues is how to correctly distinguish noise in the time-frequency domain.

Recently, we have proposed a time-frequency manifold (TFM) technique [17], which has the potential to solve the problem in TFA-based denoising approach. The TFM combines the benefits of TFA in representing the non-stationary information and manifold learning in extracting the intrinsic nonlinear structure of high-dimensional data, so it has merits in noise suppression and resolution enhancement in the time-frequency domain. The merits of the TFM benefit signal denoising based on the TFA approach [18]. We have thus proposed a TFM-based signal denoising method for a better machinery fault signal reconstruction [18]. The basic idea of this method is to synthesize a clear fault signal from the TFM signature of the raw signal. As the TFM is a time-frequency structure with a high resolution for representing impulse components of interest and excellent suppression effect for the noise, theoretically the signals reconstructed from the TFM will have satisfactory denoising effects. This paper further develops the TFM-based data denoising method in a systematic way, and addresses the utility of this method for effective fault diagnosis. Specifically, the TFM signature is learned by combining the top two TFMs in this study. The synthetic signature will have a better denoising effect in the time-frequency domain. Moreover, the TFM-based data denoising method is evaluated by introducing a clustering-based statistical parameter by considering the merit of TFM. In addition, the fault diagnosis effect of the proposed denoising method is evaluated by presenting a new diagnostic approach, called frequency probability time series (FPTS) spectral analysis. The excellent merits of TFM will be beneficial to the effectiveness of the proposed method in vibration data denoising and fault diagnosis.

In the rest of the paper, the basic TFM analysis theory of vibration signal is introduced in Section 2. In Section 3, the principle and procedure of the TFM-based data denoising method is presented, and the effectiveness of this method in denoising effects and fault diagnosis is further evaluated. Then, the effectiveness of the proposed method is verified by application to a set of practical bearing vibration sensor data in Section 4. Finally, conclusions are drawn in Section 5.

## 2. TFM Analysis of Vibration Signal

The TFM is embedded on the time-frequency distribution (TFD, which can be achieved by various TFA methods such as STFT, Continuous WT and Wigner-Ville distribution) of a non-stationary signal as an intrinsic nonlinear manifold structure in the time-frequency domain. For different vibration signals, the TFM displays different time-frequency patterns that can be extracted by a technique which addresses manifold learning on a series of TFDs in the reconstructed phase space [17]. The TFM combines non-stationary information and nonlinear information. It has a similar 2-D appearance to the TFD but possesses the advantages of noise suppression and resolution enhancement in the time-frequency domain. For details on the TFM technique readers can refer to [17]. The following provides a brief description on main steps to obtain the TFM for a vibration signal *x*(*t*).

The TFM learning requires firstly reconstructing the manifold of signal *x*(*t*) in a high-dimensional phase space by the phase space reconstruction (PSR) technique. For a signal *x*(*t*) with *N* data points, the *i*th phase point vector in an *m*-dimensional phase space is given as:

(1) $$X_{i}^{m}=\left[x_{i},x_{i+\tau },\ldots ,x_{i+(m-1)\tau }\right]$$

where *x_i_* is the *i*th data point in *x*(*t*), *m* is the embedding dimension, and *τ* is the time delay. In this study, the embedding dimension is calculated to satisfy a sufficient but not necessary condition by Cao's method [19] and the time delay is set to be one in order to keep a high time resolution [17]. The purpose of conducting PSR is to reconstruct the underlying manifold embedded in the given signal *x*(*t*) so that the manifold learning algorithm can be followed to extract the manifold. When aligning the vectors { $X_{i}^{m}|i=1,2,\ldots ,n$} in the order of time, a time-dependent data matrix *P* ϵ *R^m^* ^×^ *^n^* (*τ* = 1, *n* = *N* − *m* + 1) is constructed in the phase space with its elements having the following relationship with the data of *x*(*t*):

(2) $$P_{\left(j,k\right)}=x_{k+(j-1)\tau }$$

where *j* ϵ [1, *m*], *k* ϵ [1, *N* − (*m* − 1)*τ* ].

The TFM is then calculated in the reconstructed phase space. In this paper, the STFT is taken to generate the TFD. Firstly, each row (with the time sense) of the data matrix *P* is analyzed by the STFT to provide a time-frequency representation as shown in the following equation:

(3) $$S_{j}(k,v)=\sum_{l=-\infty }^{\infty }P_{j}\left[l\right]w\left[k-l\right]e^{-i\frac{2\pi }{M}vl},j=1,2,\cdots ,m$$

where *k* and *v* are the location of time axis and frequency axis, respectively, *M* is the discrete frequency points number in STFT, *w*(*k*) is a short-time analysis window, and *P_j_* is the *j*th row of matrix *P* with length *n*. The result *S*(*k*, *v*) is in the complex form, which can be also expressed in two parts: amplitude and phase. The amplitude part is just the TFD in this study. Therefore, *m* TFDs can be generated from the constructed data *P*. These TFDs will receive a process of frequency band selection before TFM learning to improve the computational efficiency. That is to say, only the frequency band of interest (where the main vibration pattern can be revealed) will be kept to make a reduced TFD. The *m* updated TFDs are denoted by $TFD_{x}^{m}(t,v)$ with the size of *L* × *n*, where *L* is the selected frequency points, *n* is the time points.

The updated TFDs will be the input into a manifold learning algorithm for TFM calculation. In this step, the Local Tangent Space Alignment (LTSA) algorithm [20] is employed to also calculate *d* TFMs with a size of *L* × *n* for each one. For the details of the TFM learning process, see [17]. Generally, *d* is far less than *m*. The first two TFMs are useful for representing the meaningful time-frequency signature of the vibration signal. A synthetic signature by proportionally adding these two TFMs together is successfully suggested in our previous study [17]. The synthetic TFM signature of the analyzed signal *x*(*t*) is denoted by $TFM_{x}^{s}(t,v)$ and expressed as:

(4) $$TFM_{x}^{s}(t,v)=TFM_{x}^{1}(t,v)+cTFM_{x}^{2}(t,v)$$

where $TFM_{x}^{1}(t,v)$ and $TFM_{x}^{2}(t,v)$ are the first two TFMs, *c* is a proper ratio coefficient to cancel out the noise components. The proper ratio coefficient *c* can be determined according to the noise scatter properties of the first two TFMs. For the detailed calculation principle, please refer to our previous study [17]. The calculated TFM signature has the merit that it can keep the intrinsic time-frequency structure, while the random noise can be restrained. In order to achieve a better effect, a simple zero threshold (or slightly bigger) processing can be further employed for the final TFM result.

**Figure 1.**
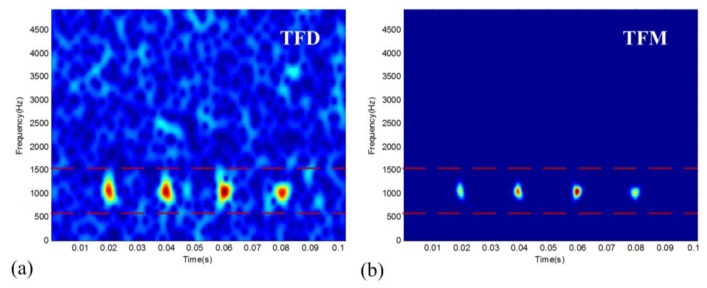
Representation of TFD and TFM for the simulated vibration signal: (**a**) TFD and (**b**) TFM.

The following provides a simulation example to demonstrate the TFM signature of the vibration signal. This example illustrates a signal with periodic transient impulses, which represents typical vibration pattern of rotating machinery. The simulated signal is constructed by considering a free vibration model with damping as follows:

(5) $$x(t)=\sum_{i=1}^{4}A(i)\exp\left\{\frac{-\zeta }{\sqrt{1-\zeta^{2}}}{\left[2\pi f_{0}(t-i\cdot p)\right]}^{2}\right\}\sin[2\pi f_{0}(t-i\cdot p)]+n_{G}(t)$$

where *f*_0_ = 1,000 Hz, *p* = 0.02 s being the impulse period, *ζ* = 0.01 being the damping ratio, *A* indicates the varying initial magnitude, and *n_G_*(*t*) is the Gaussian noise component defined by the signal-to-noise ratio (SNR). With the sampling frequency *f_s_* = 10 kHz and corruption by −5 dB white noise, the TFD of the simulated signal is shown in Figure 1a. It can be seen that the periodic transient impulses are heavily corrupted by the noise in the time-frequency domain. However, we can identify a characteristic band of the vibration signal within the range of 550–1,500 Hz. This band contains the resonance information of interest. The TFM signature (*m* = 11, *c* = 0.1) of the simulation signal is given in Figure 1b, which shows a much clearer representation of the non-stationary structure in comparison with the original TFD. The TFM signature has obvious noise suppression and resolution enhancement effects.

## 3. TFM Synthesis for Data Denoising

### 3.1. Principle and Procedure

Motivated by the merits of TFM, a new data denoising method is proposed in this study. As manifold learning can keep the intrinsic nonlinear structure in dimension reduction of a high-dimensional data matrix, the TFM signature represents the time-frequency structure nature of the original signal in the sense of noise suppression. This study introduces TFM to the signal denoising field by treating the TFM signature as a processed time-frequency result. This approach is called TFM synthesis, which mainly combines the techniques of time-frequency synthesis and PSR synthesis. With TFM synthesis, the denoised signal is expected to be reconstructed from the TFM signature. The new principle of data denoising aims to reduce background noise of signals effectively, and at the same time keep the essence of transient signals to the maximum extent. Therefore, the proposed method is especially suited for denoising faulty vibration signals.

In the principle of TFM synthesis, the TFM signature result is used to replace each of *m* selected TFDs in the phase space while keeping the relative amplitude. Here keeping relative amplitude is to make the peak values in the TFD and the TFM equal. At the same time, the part of TFD that is not selected for manifold learning is set to be zero. In this way, we can re-generate *m* TFDs in the entire frequency band. Combining the re-generated *m* TFDs with the *m* original phase part of *S_j_*(*k*, *v*), *j* =1,2, …, *m*, then *m* updated STFT results, denoted by *Ŝ_j_*(*k*, *v*), *j* = 1,2, …, *m*, can be generated.

Then time-frequency synthesis is employed on each updated STFT result to calculate a new data matrix *P̂* in the phase space. The time-frequency synthesis of STFT can be expressed as follow:

(6) $${\hat{P}}_{j}[k]=\frac{1}{Mw[0]}\sum_{v=0}^{M-1}{\hat{S}}_{j}(k,v)e^{i\frac{2\pi }{n}kv},j=1,2,\cdots ,m$$

Assume *w*[*n*] (with the window band width *w_c_* and the window length *N_w_*) is not equal to zero in the limited window length, then Equation (6) holds under the following constraints [14]: (a) the time sampling factor *L* of STFT should satisfy the Nyquist criteria, that is $L\leq \frac{2\pi }{w_{c}}$; (b) the frequency sampling interval should satisfy $\frac{2\pi }{N}\leq \frac{2\pi }{N_{w}}$, that is *N* ≥ *N_w_*. As each STFT result can generate a time series by time-frequency synthesis, *m* time series could thus construct a data matrix *P̂* with the same size as original data matrix *P*.

After getting the updated data matrix *P̂* in the phase space, the PSR synthesis is applied to reconstruct the signal with denoising effect. In the process of reconstruction, it should be considered that every element of the original time series may appear at several places in the phase space data matrix. The PSR synthesis is presented as follows to reconstruct the signal from data matrix *P̂*:

(7) $${\hat{x}}_{i}=\frac{\sum_{q\in \left\{I_{i}(j,k)\right\}}{\hat{P}}_{q}}{C_{i}},i=1,2\ldots ,N;j=1,2\ldots ,m$$

where {*I_i_*(*j*, *k*) } is the subscript set of the signal elements that meets the requirement of *k* + (*j* − 1)*τ* = *i* (*k* ϵ [1, *N* − (*m* − 1)*τ* ]), and *C_i_* is the number of elements in {*I_i_*(*j*, *k*)}. The final result of the denoised signal can be thus represented as *x̂*(*t*) with *N* data points.

In summary, the procedure of the proposed data denoising method can be described briefly as follows:

(1) Given a signal *x*(*t*) with *N* data points, calculate the data matrix *P* of size *m* × *n* (*n* = *N* − *m* + 1) by PSR according to Equation (2).
(2) Do the STFT for each row of matrix *P* via Equation (3) to get *S_j_*(*k*, *v*), *j* = 1,2, …, *m*, and calculate the corresponding amplitude and phase parts.
(3) Select the frequency band of interest to get *m* TFDs with the size of *L* × *n*.
(4) Calculate the TFM of size *L* × *n* by manifold learning and the synthetic TFM signature via Equation (4), and conduct zero or slightly bigger value thresholding for the TFM signature.
(5) Update the STFT result using original phase and the element-adjusted TFM signature as new amplitude to get *Ŝ_j_*(*k*, *v*), *j* = 1,2, …, *m*.
(6) A new data matrix *P̂* of size *m* × *n* in phase space is generated by time-frequency synthesis according to Equation (6).
(7) The denoised signal *x̂*(*t*) is finally reconstructed by PSR synthesis by Equation (7).

### 3.2. Evaluation

**Figure 2.**
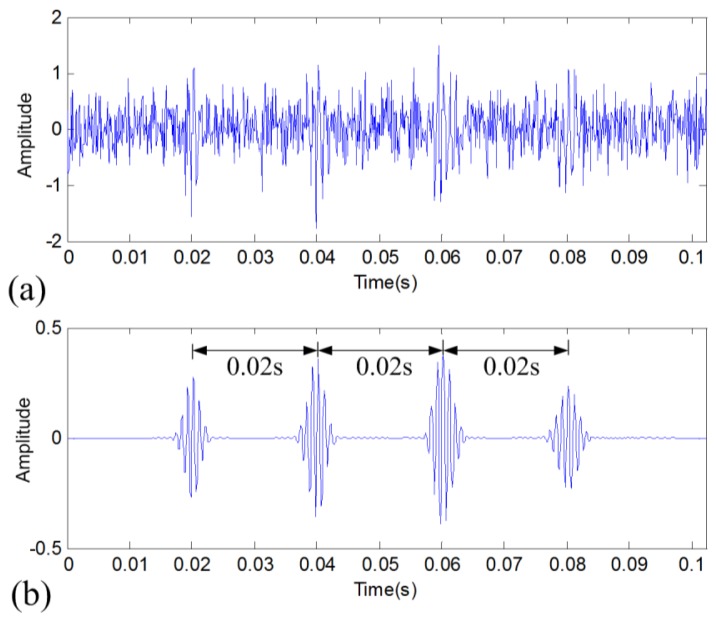
Result of the proposed TFM-based data denoising method for the simulated signal: (**a**) original noisy signal and (**b**) denoised signal.

**Figure 3.**
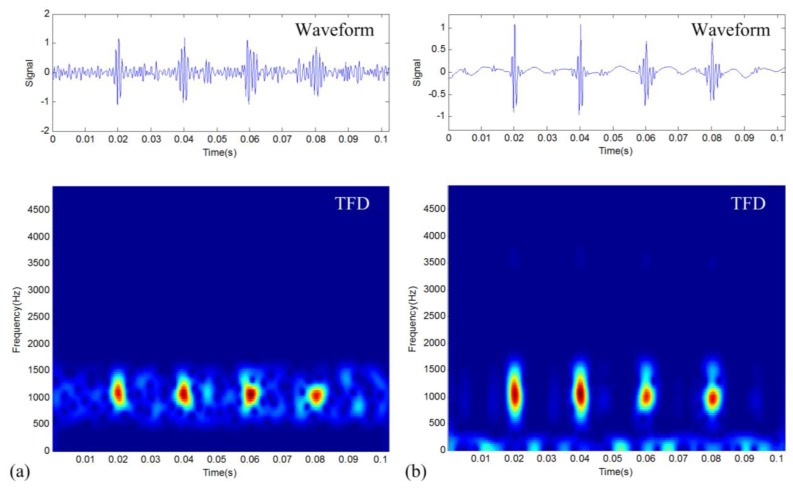
Denoising results of two traditional methods: (**a**) band-pass filtering and (**b**) DWT-based denoising.

To verify the effectiveness of the proposed data denoising method, the simulated vibration signal provided above is further analyzed for signal denoising. By using the proposed method, the reconstructed signal shown in Figure 2b is obtained. Compared to the original noisy signal shown in Figure 2a, it can be found that the reconstructed signal shows an excellent denoising effect as the periodic transient impulses are clearly captured, while the noise is well discarded. Now the period of the impulses can be well estimated as marked in Figure 2b. To confirm the superiority of this result, two other traditional methods are also employed to analyze this signal for comparison. They are band-pass filtering method and discrete wavelet transform-based (DWT-based) thresholding denoising method. The results are displayed in Figure 3. The result in Figure 3a still contains obvious noise as compared to original signal as the in-band noise is still very heavy. Although the result in Figure 3b has a certain denoising effect, it does not retain the natural impulse characteristics of the original raw signal and has bad frequency resolution. By comparing the three denoising methods, it can be found that the denoising effect of the proposed method is obviously superior to the other two methods.

To further evaluate the result of the proposed method quantitatively, a new parameter is proposed as follows to assess the quality of signal denoising in the context of machinery fault diagnosis. This parameter considers the sparse property of impulses in time. As the proposed data denoising method keeps the merits of TFM signature, its result will deliver the natural transient waveform according to the well-captured time-frequency structure. The clean periodic impulsive signal considered in this paper will indicate good sparse property in the time domain. This property will be greatly beneficial to machinery fault diagnosis. Thus we propose a statistical parameter, called clustering statistical parameter (CSP), to describe the time-clustering property of periodic impulses. The CSP is calculated by the following procedure:

*Step 1*: Pre-process an analyzed signal by using mean-variance standardization and convert its absolute amplitude to binary representation with values 0 or 1 using a slight threshold.
*Step 2*: Generate a sampling point index vector of the analyzed signal and conduct its dot product with the binary time series produced in Step 1. Discard the zero elements and only keep the positive ones, resulting in a new vector.
*Step 3*: Conduct the *k*-means clustering for the new vector produced in Step 2 by setting the class number to be the number of impulses contained in the analyzed signal.
*Step 4*: Calculate the between-class distance *D_b_* and the within-class distance *D_w_* of the clusters (for calculation of these two distances, please refer to [21,22]) and then calculate their ratio *D_b_*/*D_w_*. This is the final CSP value.

To demonstrate the effectiveness of the new parameter, a simulation is conducted to show the relationship between the CSP and the SNR parameters. Specifically, the simulated signal as expressed in Equation (5) is generated by decreasing the SNR values according to Table 1. Then the presented parameter is calculated on these signals with different SNR conditions. The result is shown in Table 1, where it can be seen that the parameter value reduces when the SNR decreases. Since the decrease of the SNR corresponds to the degradation of the waveform quality, the parameter could provide a quantitative tool for characterizing the quality of signal denoising.

Except for the relation with SNR, the CSP can also characterize well the sparse property of an ideal periodic impulsive signal, which is directly beneficial to machinery fault diagnosis. The CSP parameter is further calculated for the results of three denoising methods. As shown in Table 2, it can be seen that the proposed TFM-based method shows the best result as compared to the other methods, which indicates its benefits in characterizing the sparse periodic impulses. This benefit will be exhibited later to show that it can help improve fault diagnosis.

### 3.3. Fault Diagnosis

In machinery fault diagnosis, the main aim is to effectively identify the fault physics based on vibration signal analysis. The vibration signal from a rotating machine with a localized defect generally presents a periodic transient impulse property just as illustrated in the above simulation signal. As the measured vibration signal will also contain background noise coming from the working environment, signal filtering or denoising is necessary for effectively diagnosing the specific machinery fault. The previous section verifies the effectiveness and the superiority of the proposed method for data denoising. The following will validate the benefit of the TFM-based data denoising method for effective fault diagnosis.

**Figure 4.**
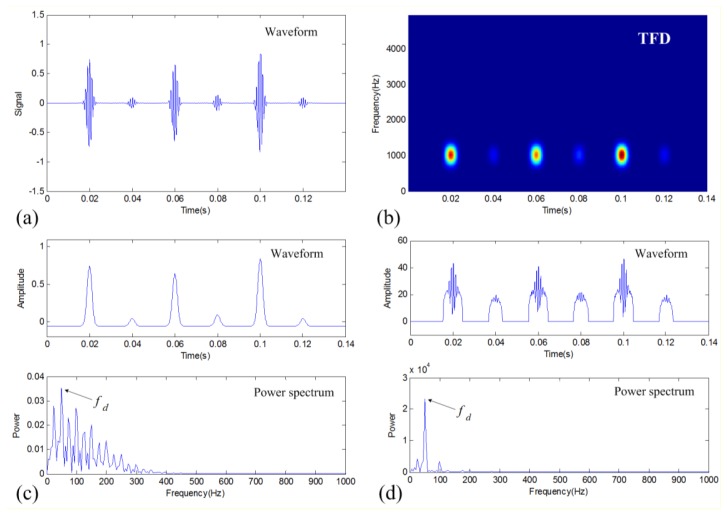
Demonstration of the FPTS spectral analysis for a simulated signal with periodic impulses of different intensities: (**a**) waveform; (**b**) the TFD; (**c**) envelope spectrum; and (**d**) FPTS spectrum.

The above CSP parameter indicates that the proposed denoising method can capture well the fault-related sparse periodic impulses. However, this parameter doesn't consider the difference of each impulse intensity. A general fault diagnosis approach, the envelope spectral analysis, will have worse performance when the impulses have a bigger difference in amplitude. A demonstration using a simulated signal with periodic impulses of different intensities is shown in Figure 4, where it can be seen that the envelope spectrum is not very powerful to identify the characteristic frequency *f_d_*. However, in practical vibration fault signals, this condition is commonly seen. Considering this, it is necessary to present a new method to improve the performance of fault diagnosis. This paper thus proposes a new method that utilizes the time-frequency structure property according to the merit of TFM. The method just considers the area of the impulses and ignores their intensity information in the time-frequency representation. It is called frequency probability time series (FPTS) spectral analysis, which is mainly realized through the following procedure:

*Step 1*: Convert the intensity TFD matrix to a binary TFD matrix by using a threshold. The thresholding operation is used to remove the effect of random noise, so the threshold value is related to the noise level. Less the noise level is, smaller the threshold is taken. Generally, we can take the value as ten percent to twenty percent of the peak value in the TFD matrix.
*Step 2*: The binary TFD matrix is summarized along frequency so that a time series reflecting the frequency probability information of impulses is achieved.
*Step 3*: The spectral analysis is finally conducted on the new time series and the fault characteristic frequency can thus be identified.

Different from the traditional approach that considers the intensity information of impulses, this FPTS-based diagnostic approach considers the distribution information of impulses in the time-frequency domain. The performance of this proposed method is demonstrated in Figure 4d. It can be seen that the new diagnostic approach can well utilize the time-frequency structure information of the periodic transient impulses to improve the diagnostic effect.

**Figure 5.**
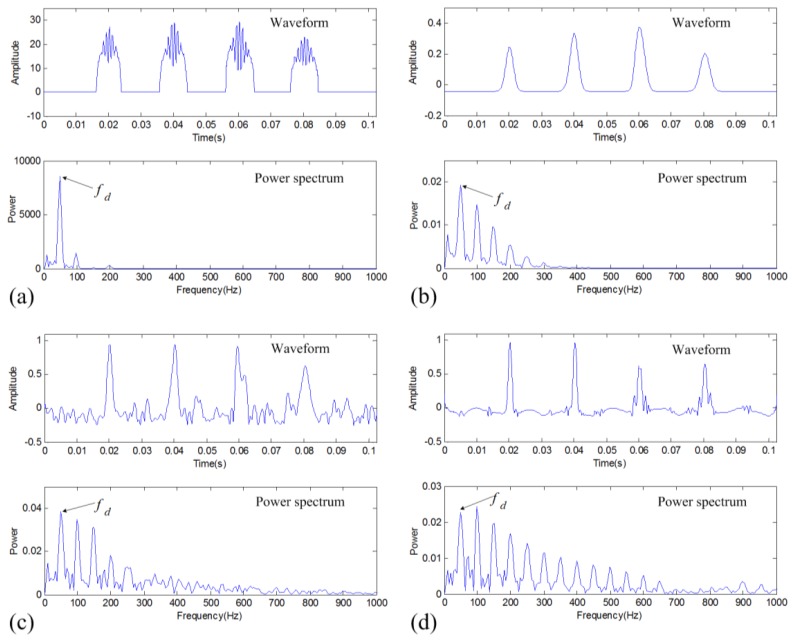
Fault diagnosis results of the simulation case for different methods: (**a**) FPTS spectrum of the TFM-based denoising result; (**b**) envelope spectrum of the TFM-based denoising result; (**c**) envelope spectrum of band-pass filtering result; and (**d**) envelope spectrum of DWT-based denoising result.

Using the proposed FPTS spectral analysis method, the TFM-based denoising result is further analyzed as shown in Figure 5a. It can be seen that the fault characteristic frequency *f_d_* = 50 Hz is clearly identified in the final spectrum. To verify the effectiveness of the FPTS-based fault diagnosis method, the traditional envelope spectral analysis is also conducted on the denoising results of three different methods for comparison. These envelope analysis results are shown in Figure 5b–d for the TFM-based denoised signal, band-pass filtered signal, and DWT-based denoised signal, respectively. As compared to these results, the FPTS result indicates the merits of clear impulsiveness and similar intensity for each impulses, so its spectrum absolutely displays the fault characteristic frequency with the least interference of other frequency components. Therefore, the FPTS of the TFM-based denoising result is greatly beneficial to machinery fault diagnosis.

## 4. Experimental Verification

**Figure 6.**
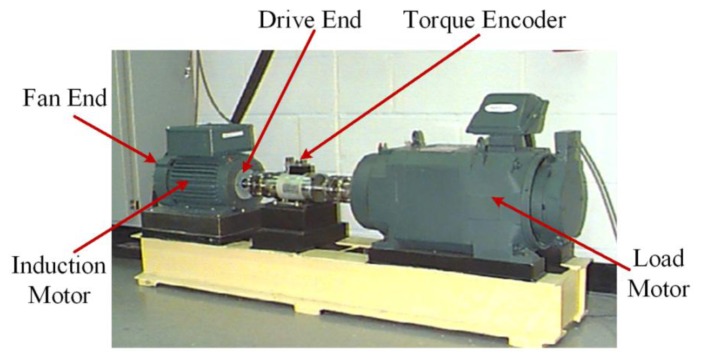
The bearing test stand.

To verify the effectiveness of the proposed TFM-based denoising method in practical engineering applications, a set of bearing data with rolling-element defect, inner-race defect and outer-race defect are analyzed. The above two traditional denoising methods are also employed for a comparison. The set of bearing data were acquired by using an experimental setup as shown in Figure 6 from the Case Western Reserve University (CWRU) Bearing Data Center [23]. The test stand consists of a 2 hp motor (left), a torque transducer/encoder (center), a dynamometer (right), and control electronics (not shown). The motor shaft is supported by the test bearings. Vibration data were collected using accelerometers attached to the housing with magnetic bases, at a sampling frequency of 12 kHz for driving end bearing experiments. The test bearings are deep groove ball bearings of the 6205-2RS JEM SKF type. Single point defects were set on the test bearings separately at the rolling element, inner raceway and outer raceway using electro-discharge machining. Accelerometers were placed at the 12 o′clock position when the defects were at the rolling element and inner raceway, and at the 6 o'clock position for the outer raceway defect. The parameters on the three signals to be analyzed are listed in Table 3.

### 4.1. Rolling-Element Defective Bearing Vibration Data

**Figure 7.**
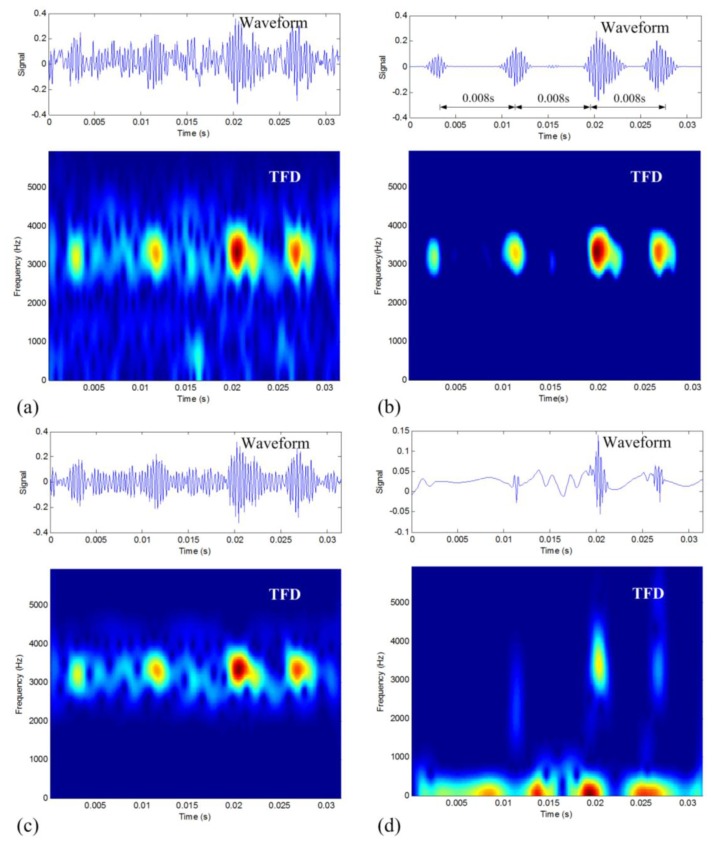
Denoising results of the rolling-element defective vibration signal: (**a**) original raw signal; (**b**) the TFM-based denoising result; (**c**) band-pass filtering result; and (**d**) DWT-based denoising result.

The vibration signal with rolling-element defect is analyzed first. The waveform and the TFD of the defective signal are shown in Figure 7a. It can be seen that the waveform indicates a series of similar periodic impulses along time, but the impulse period cannot be identified from the waveform as there is noise corruption. The TFD presents a combination of time and frequency information. It can be seen that there is a series of impulses along the 3,200 Hz line. However, there also exists the issue of noise corruption.

The proposed TFM-based data denoising method is then applied to the signal. The selected frequency band of interest is from 2,200 Hz to 4,200 Hz. To improve computational efficiency, the parts of the obtained *m* TFDs (by the PSR with *m* = 11) in the selected frequency band are kept. According to the procedure presented in Section 2, the TFM signature is achieved by taking *c* = −0.4 and displayed in Figure 7b. By comparing Figure 7a,b, it can be seen that noise corruption in Figure 7b is greatly improved as compared to that in Figure 7a. Importantly, the original time-frequency structure of the impulses is very well kept in the TFM signature. By the time-frequency synthesis and PSR synthesis according to Section 3.1, the final denoised waveform is shown in Figure 7b, which presents a series of periodic impulses with minimal noise. It can be estimated that the average time period of these impulses is around 0.008 s, which is close to the theoretical value of 0.0071 s. The result confirms that the proposed denoising method can reduce noise effectively and keep the natural structure of fault signals.

As a comparison, the results of band-pass filtering method and DWT-based denoising method are displayed as Figure 7c,d, respectively. The result in Figure 7c has almost no noise reduction effect as compared to the original signal since there is plenty of in-band noise. As seen in Figure 7d, the DWT-based denoising result misses the weakest impulse and it does not keep the original impulse characteristics of the raw signal. By comparing the four results in Figure 7, it can be found the denoising effect of the proposed method is obviously superior to the other two methods. To quantitatively describe this difference, the presented CSP values are calculated for these three results as listed in Table 4. In this quantitative way, we can also find that the proposed TFM-based denoising method shows the best result.

**Figure 8.**
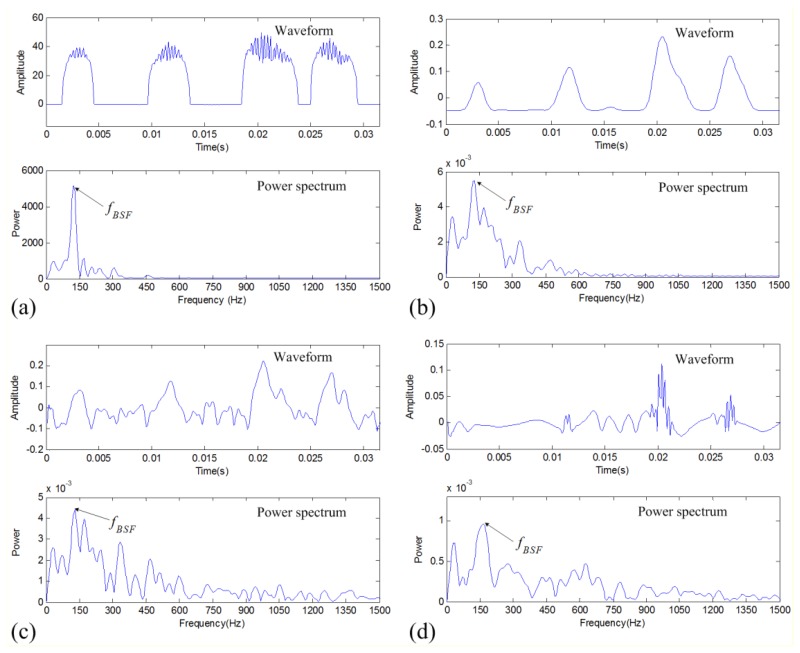
Fault diagnosis results of rolling-element defective vibration signal: (**a**) FPTS spectrum of TFM-based denoised signal; (**b**) envelope spectrum of TFM-based denoised signal; (**c**) envelope spectrum of band-pass filtered signal; and (**d**) envelope spectrum of DWT-based denoised signal.

To demonstrate the effectiveness of the proposed data denoising method for fault diagnosis, the denoising results are further analyzed. The TFM-based denoised signal is analyzed by the FPTS spectral analysis and the envelope spectrum analysis methods, respectively, as shown in Figure 8a,b.

It can be seen that the FPTS shows similar intensities for different impulses, which contributes an absolute defective frequency component in the spectrum. However, in the envelope spectrum of the TFM-based denoising result, there exist other obvious frequency components except the defective frequency *f_BSF_*. For comparison, the denoised signals of two traditional methods are also analyzed by the envelope spectral analysis. The results are indicated in Figure 8c,d, respectively. It can be found that they show worse results than the TFM-based denoising result in both the envelope waveform and envelope spectrum. Therefore, the FPTS spectrum of the TFM-based denoised signal shows the best diagnostic effect for the rolling-element defect.

### 4.2. Inner-Race Defective Bearing Vibration Data

**Figure 9.**
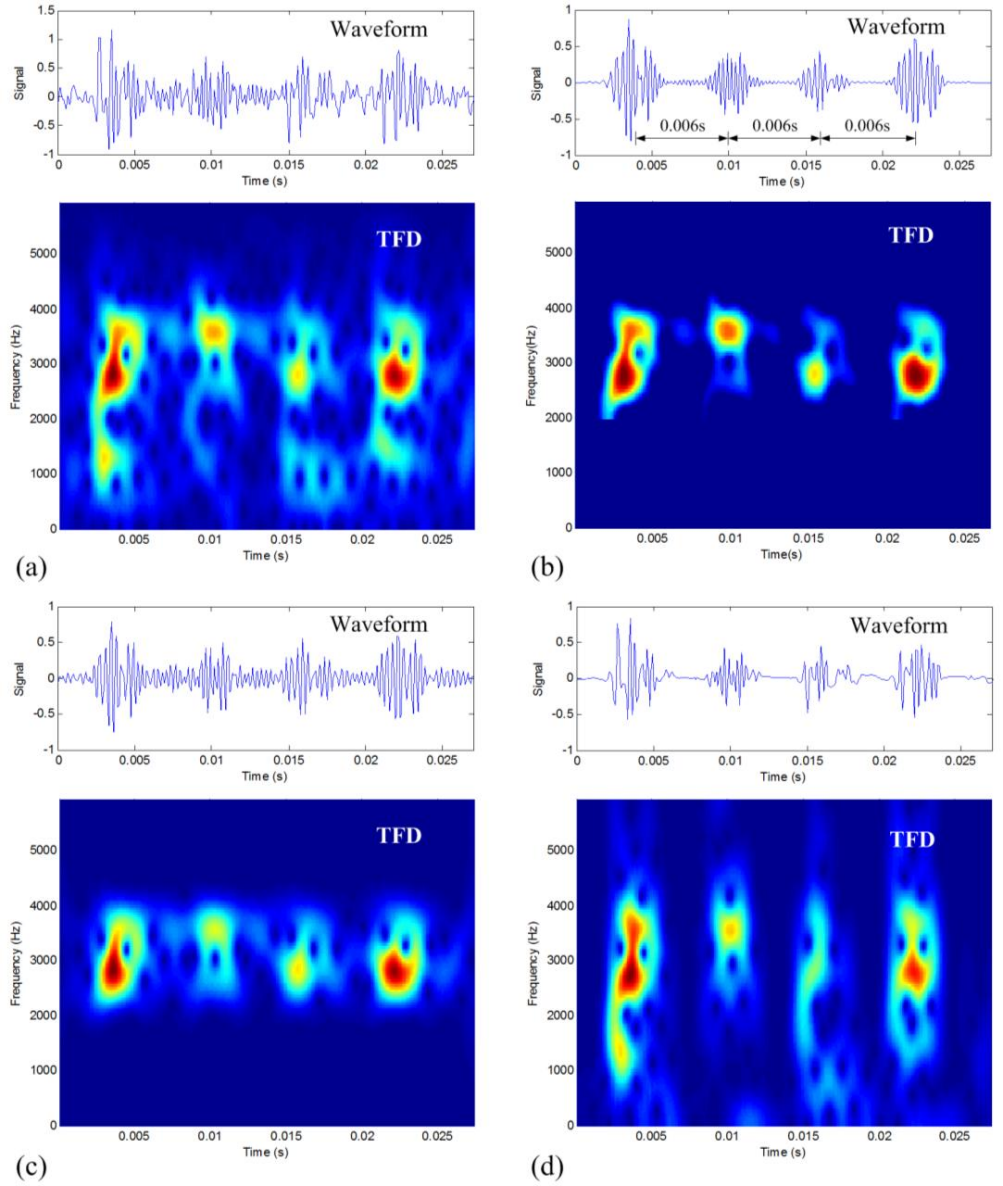
Denoising results of the inner-race defective vibration signal: (**a**) original raw signal; (**b**) the TFM-based denoising result; (**c**) band-pass filtering result; (**d**) DWT-based denoising result.

The vibration signal with inner-race defect is shown in Figure 9a. It can be seen that the waveform indicates a series of similar periodic impulses buried in the noise. The TFD presents a combination of time and frequency information. It can be found that there exists the issue of noise corruption in a wide frequency band.

To remove noise, the proposed TFM-based data denoising method is then applied to the signal. In the TFM signature calculation, the 2,000–4,300 Hz frequency band of interest, is selected to improve computational efficiency. The obtained TFM signature (*m* = 11, *c* = −0.1) is displayed in Figure 9b. Compared to Figure 9a, it can be seen that the level of in-band noise corruption in Figure 9b is greatly improved, and at the same time, the original time-frequency structure of the impulses is very well retained in the TFM signature. After the time-frequency synthesis and the PSR synthesis, the final denoised waveform is shown in the waveform of Figure 9b, which clearly presents a series of periodic impulses with very good sparse property in time. It can be estimated that the average time period of these impulses is around 0.006 s, which is very close to the theoretical value of 0.0062 s. The result confirms that the proposed denoising method can keep the original impulse structure of fault signals while reducing noise.

For comparison, the results of the other two traditional methods are displayed as Figure 9c,d, respectively. It can be seen that band-pass filtering keeps good time-frequency structure of the impulses, however, the in-band noise is also kept so the noise is still obvious in the waveform. The DWT-based denoising result identifies well the location of impulses, but the time-frequency structure of the impulses is distorted as compared to the TFM result, and the out-of-band noise is still retained. In the four results in Figure 9, it can be found the proposed method shows the best denoising effect. To quantitatively describe this difference, the presented CSP values are also calculated for these results as listed in Table 5, where we can also find that the proposed TFM-based denoising method is the best in the sense of sparse property preserving for the periodic impulses.

**Figure 10.**
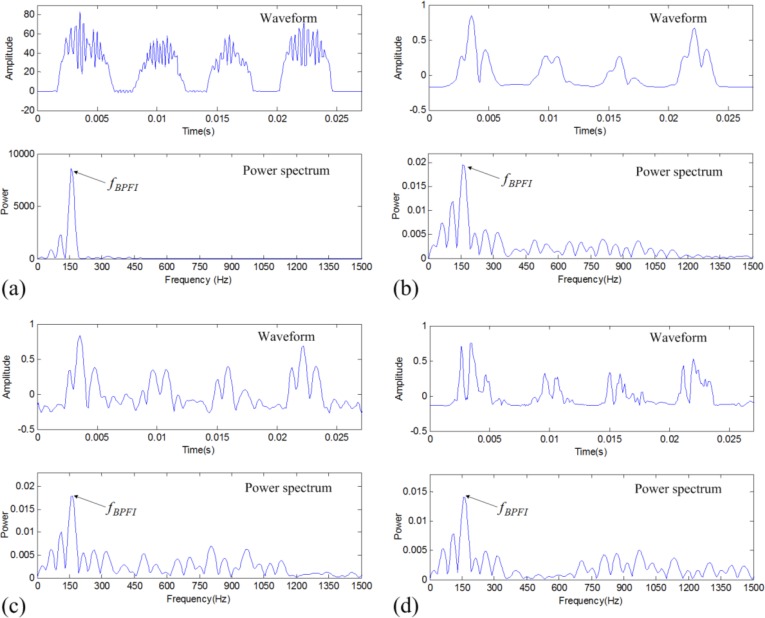
Fault diagnosis results of the inner-race defective vibration signal: (**a**) FPTS spectrum of TFM-based denoised signal; (**b**) envelope spectrum of TFM-based denoised signal; (**c**) envelope spectrum of band-pass filtered signal; and (**d**) envelope spectrum of DWT-based denoised signal.

The above denoising results are further analyzed to verify the effectiveness of the proposed data denoising method for fault diagnosis. The FPTS spectrum and envelope spectrum of the TFM-based denoised signal are shown in Figure 10a,b, respectively. It can be seen that the FPTS shows impulses with similar intensities and time widths, so its spectrum shows rather clearer the defective frequency component *f_BPFI_* than the envelope spectrum. For comparison, the denoised signals by two traditional methods are also analyzed using the envelope spectral analysis method as indicated in Figure 10c,d, respectively. These two results show more interfering noise components than the TFM-based result. This verifies that the TFM-based denoising result has good sparseness for periodic impulses and the FPTS is powerful in improving fault diagnosis.

### 4.3. Outer-Race Defective Bearing Vibration Data

**Figure 11.**
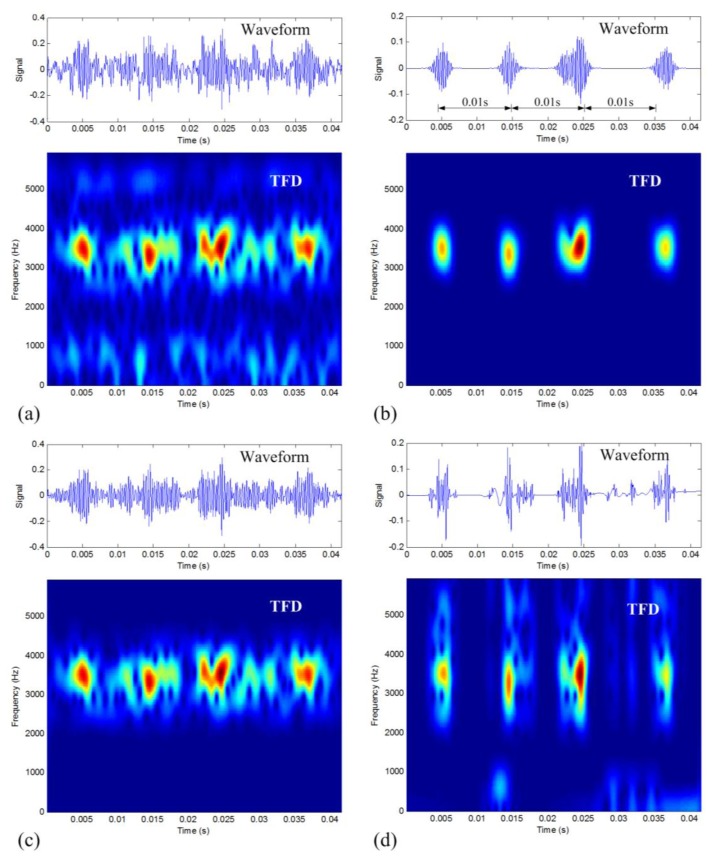
Denoising results of the outer-race defective vibration signal: (**a**) original raw signal; (**b**) the TFM-based denoising result; (**c**) band-pass filtering result; and (**d**) DWT-based denoising result.

The outer-race defective vibration signal is analyzed to verify the proposed method. Figure 11 shows the waveform and the TFD of the signal. It can be seen that the existence of noise in the waveform makes it hard to discern the nature of the signal. From the TFD, we can see a series of impulses along the 3,500 Hz frequency line, but the existence of strong background noise makes it difficult to identify the period of these impulses. Moreover, we can determine the frequency band of interest as 2,100–4,600 Hz from the TFD to calculate the TFM signature. Based on the TFM signature (*m* = 11, *c* = 0.1), the synthesized denoised signal and its TFD are displayed in Figure 11b. It can be seen that the impulses are much clearer than those in Figure 11a with the in-band noise being very well removed. Now the average time period of these impulses can be estimated to be around 0.01 s, very close to the calculated theoretical value of 0.0096 s. Therefore, the proposed denoising method is also verified to be able to reduce noise effectively, as well as to keep the nature of fault signals.

Two traditional denoising methods, band-pass filtering method and DWT-based denoising method, are also used to process this signal. The results are displayed in Figure 11c,d, respectively. By comparing these four pictures in Figure 11, the denoising effect of the proposed method is proved to be much better than the traditional methods. The calculated CSP values as listed in Table 6 also quantitatively confirm that the proposed TFM-based method is the most effective for data denoising as well as impulse sparseness keeping.

**Figure 12.**
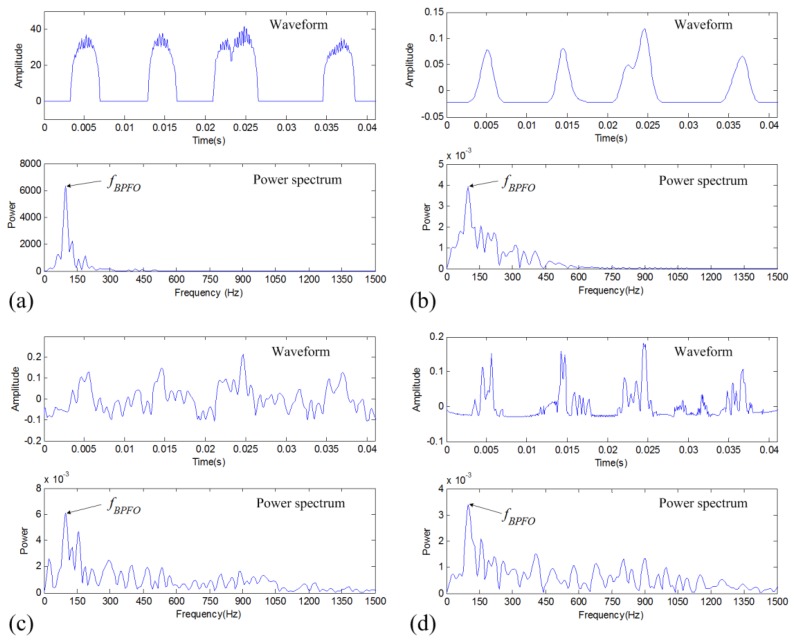
Fault diagnosis results of the outer-race defective vibration signal: (**a**) FPTS spectrum of TFM-based denoised signal; (**b**) envelope spectrum of TFM-based denoised signal; (**c**) envelope spectrum of band-pass filtered signal; and (**d**) envelope spectrum of DWT-based denoised signal.

The above denoising results are again analyzed to verify the fault diagnosis effectiveness of the proposed method. As seen in Figure 12a,b, the FPTS spectrum of the TFM-based denoised signal shows a rather clearer spectrum to identify the defective frequency component *f_BPFO_* than its envelope spectrum. For comparison, the denoised signals obtained by two traditional methods are also analyzed using the envelope spectral analysis method. As indicated in Figure 12c,d, it can be seen that the band-pass filtering result doesn't show the impulsive signature clearly and the DWT-based denoising result indicates more interfering noise components. Therefore, the TFM-based denoising and its FPTS analysis show great benefits for machinery fault signature analysis and diagnosis.

## 5. Conclusions

This paper presents a novel vibration sensor data denoising method which employs TFM to reconstruct a clean signal from the noisy raw signal by combining techniques of time-frequency synthesis and PSR synthesis. Based on the discussions above, following conclusions can be drawn:

(1) The proposed denoising method inherits the merits of TFM in noise suppression and resolution enhancement to represent an intrinsic time-frequency structure, so it does not only reduce background noise effectively, but also keeps the intrinsic time-frequency structure of the periodic transient impulses in rotating machinery fault signal, which is significant for intrinsic vibration data characteristics and reliable fault diagnosis.
(2) The proposed FPTS spectral analysis method is beneficial to utilizing the good sparse property of the TFM in impulse characteristics, and hence can be combined well with the proposed data denoising method for an improved fault diagnosis.
(3) The performance of the proposed data denoising method and its FPTS spectral analysis has been verified by means of defective bearing data in comparison with the band-pass filtering and the DWT-based denoising methods. The proposed method shows great benefits and value in vibration sensor data denoising for effective machinery fault diagnosis.

## Figures and Tables

**Table 1.** The relationship between the CSP and the SNR.

**SNR (dB)**	+∞ (Clean signal)	30	20	10	0	−5	−∞ (White noise)
**CSP**	0.3021	0.0774	0.0399	0.022	0.018	0.0172	0.0168

**Table 2.** The CSP values for the denoising results of different methods.

**Denoising Method**	**Proposed Method**	**Band-Pass Filtering**	**DWT-Based Denoising**
CSP	0.2268	0.0193	0.0237

**Table 3.** Parameters of tested bearings.

**Defect Type**	**Defect Size** **(D × W) (in)**	**Rotation Speed** **(rpm)**	**Fault Characteristic** **Frequency (Hz)**
Rolling-Element Defect	0.011 × 0.021	1,796	*f_BSF_* = 141.1
Inner-Race Defect	0.011 × 0.007	1,797	*f_BPFI_* = 162.2
Outer-Race Defect	0.011 × 0.014	1,749	*f_BPFO_* = 104.5

**Table 4.** The CSP values for the denoising results of different methods for rolling-element defective signal.

**Denoising Method**	**Original Signal**	**Proposed Method**	**Band-Pass Filtering**	**DWT-Based Denoising**
CSP	0.0441	0.1893	0.0458	0.049

**Table 5.** The CSP values for the denoising results of different methods for inner-race defective signal.

**Denoising Method**	**Original Signal**	**Proposed Method**	**Band-Pass Filtering**	**DWT-Based Denoising**
CSP	0.0617	0.1587	0.0619	0.1137

**Table 6.** The CSP values for the denoising results of different methods for outer-race defective signal.

**Denoising Method**	**Original Signal**	**Proposed Method**	**Band-Pass Filtering**	**DWT-Based Denoising**
CSP	0.0381	0.2693	0.0413	0.1303
